# Genetic diversity and population genetics of the warble flies *Hypoderma bovis* and *H. sinense* in Qinghai Province, China

**DOI:** 10.1186/s13071-016-1416-6

**Published:** 2016-03-12

**Authors:** Yong Fu, Wei Li, Hong Duo, Zhi-Hong Guo, Ying Li, Yan-Ming Zhang

**Affiliations:** College of Veterinary Medicine, Northwest A & F University, Yangling, Shaanxi 712100 China; Academy of Animal and Veterinary Medicine, Qinghai University, Xining, Qinghai 810016 China; College of Agriculture and Animal Husbandry, Qinghai University, Xining, Qinghai 810016 China

**Keywords:** *Hypoderma bovis*, *Hypoderma sinense*, Hypodermosis, Cytochrome *c* oxidase I, Population genetics

## Abstract

**Background:**

*Hypoderma bovis* and *H. sinense* (Diptera: Oestridae) mainly parasitise cattle and yaks. The two parasites are pathogenic and cause economic losses that result from reduced amounts of livestock products, including milk, meat, and skin. Genetic diversity and population genetic structure of *H. bovis* and *H. sinense* have not been evaluated, but could be used to inform appropriate strategies to control these parasites.

**Methods:**

We cloned and sequenced part of the mitochondrial cytochrome *c * oxidase subunit I (COI) gene from 60 *H. bovis* isolates and 52 *H. sinense* isolates from five locations in Qinghai Province, China, to identify polymorphisms, and infer their phylogenetic relationships, historical population expansions, and divergence time.

**Results:**

We identified 17 COI haplotypes from the *H. bovis* samples, and 23 COI haplotypes from the *H. sinense* samples. The haplotype and nucleotide diversities were 0.738 and 0.00202 for *H. bovis*, and 0.867 and 0.00300 for *H. sinense*, respectively, which indicates rich genetic diversity in *H. bovis* and *H. sinense* populations. Bayesian phylogenetic analysis revealed that the two species are monophyletic, and geographical structuring of haplotypes was significantly different in *H. sinense* (*P* < 0.05), but not *H. bovis*. Neutrality tests and mismatch distribution statistical analysis revealed that populations of the two species have undergone demographic expansions. The divergence three *Hypoderma* spp. (*H. bovis*, *H. lineatum*, and *H. sinense*) was estimated to have occurred approximately 4.5 million years ago (Mya), which indicates that the rapid uplift of the Qinghai-Tibetan Plateau during the late Miocene-Pliocene was associated with divergence of *Hypoderma* species.

**Conclusions:**

Results of the present study revealed that both *H. bovis* and *H. sinense* displayed high genetic diversity and widespread population genetic differentiation within and among populations; these data, along with the molecular phylogeny, demographic history, and divergence time estimation, provide new insight into evolutionary history of these species. These findings will help elucidate speciation in *Hypoderma* and provide theoretical basis for epidemiological surveillance and control of these species on the Qinghai-Tibetan Plateau.

## Background

*Hypoderma bovis* and *H. sinense* (Diptera: Oestridae) are two species of flies in Oestridae and mainly parasitize cattle and yaks. The parasitizing flies are widely distributed in north and southwestern China [1, 2]. The prevalence of *Hypoderma* spp. larval infection in yaks can reach up to 100 % in some areas of Qinghai Province [3]. Hypodermosis of cattle and yaks, caused by the larvae of *Hypoderma* spp. , is responsible for substantial economic losses in the livestock industry because it results in spontaneous abortion, reduced milk production, loss of weight, reduced fertility, and poor hide quality [4, 5]. Therefore, there is a need to develop effective strategies to control this disease.

The mitochondrial cytochrome *c *oxidase subunit I (COI) gene is a molecular marker [6, 7] used for taxonomic differentiation [8, 9], molecular identification [10, 11], and evolutionary studies [12, 13]. Understanding the genetic diversity and population structure of pests are crucial for developing effective management strategies [14].

Therefore, in the present study, we analysed the genetic diversity of *H. bovis* and *H. sinense* based on mitochondrial COI sequences in samples collected from Qinghai Province, China. In addition, we investigated possible historical population expansions and divergence time of *H. bovis* and *H. sinense*. These findings are essential for understanding speciation of *Hypoderma* spp.  and for epidemiological surveillance and control of these species on the Qinghai-Tibetan Plateau (QTP).

## Methods

### Locations

**Fig. 1:**
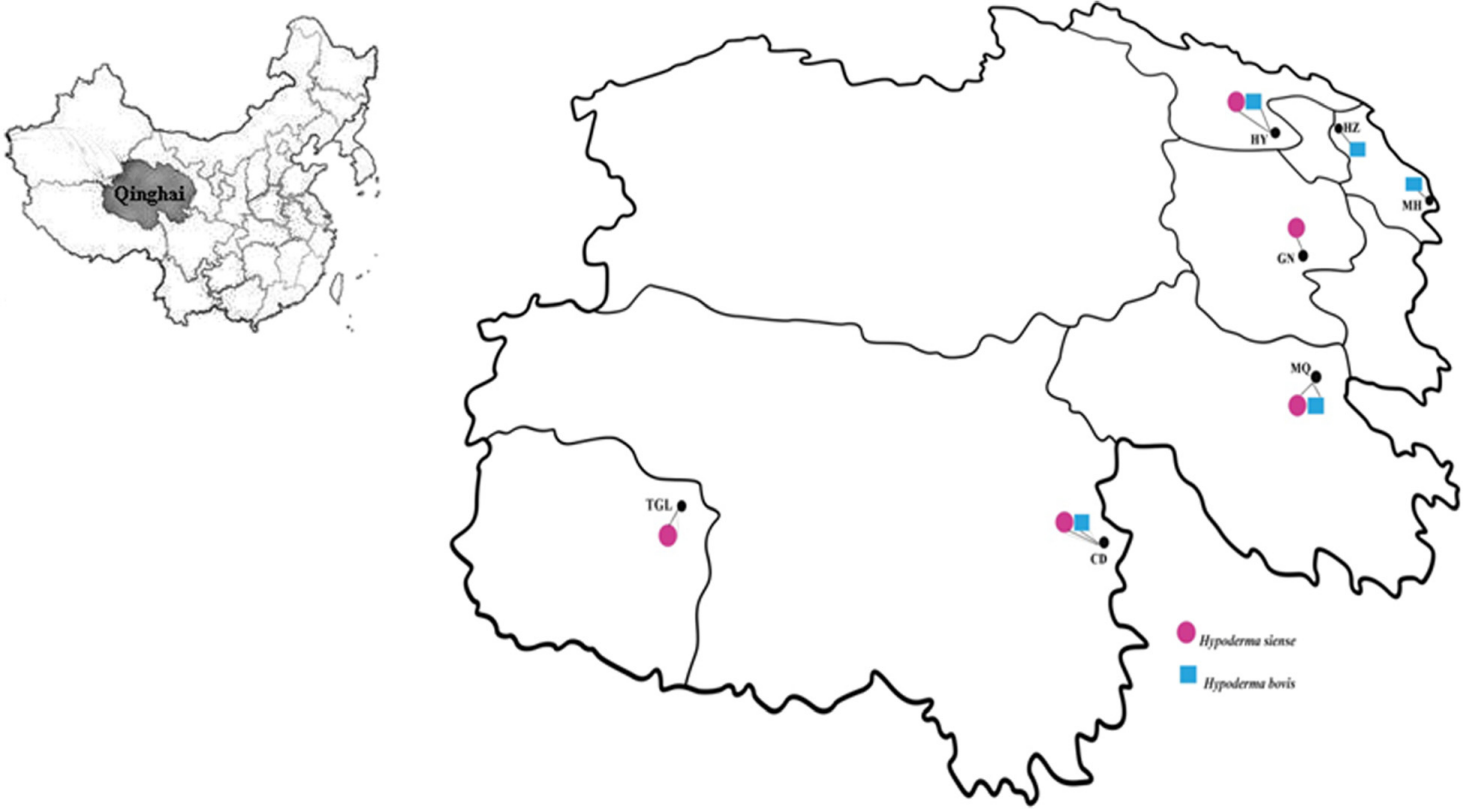
Collection sites of *H. bovis* and *H. sinense* from Qinghai province of China. Population codes correspond to those in Table 1

*Hypoderma bovis* and *H. sinense* were sampled from five localities in Qinghai Province, located in the northeastern part of the QTP in western China. The province covers a total area of over 721,000 km^2^, spanning approximately 1200 km east–west and 800 km north–south, with an average elevation higher than 3000 m above sea level [15, 16]. For *H. bovis*, sampling localities were in Maqin county (MQ), Haiyan county (HY), Huzhu county (HZ), Minhe county (MH), and Chengduo county (CD). For *H. sinense*, sampling localities were in Maqin county (MQ), Guinan county (GN), Haiyan county (HY), Tanggula town (TGL), and Chengduo county (CD) (Fig. 1).

### Sampling strategy

We collected 60 third-stage *H. bovis* larvae and 52 third-stage *H. sinense* larvae from five localities in Qinghai Province from 2013 to 2014 (Fig. 1). The initial identification of *H. bovis* and *H. sinense* was mainly based on morphological characteristics [17], and confirmed by molecular methods using the mitochondrial COI gene [8]. All specimens were fixed by immersion in 70 % ethanol. The locations and sample numbers of *H. bovis* and *H. sinense* populations are shown in Table 1.

**Table 1:** Summary statistics observed in *H. bovis* and *H. sinense* populations in this study

Species	Collection site	Population code	n	NH	*h*	π	Haplotype frequency	GenBank accession number
*H. bovis*			60	17	0.738	0.00202		
	Maqin county	MQ	13	8	0.923	0.00350	HB1(3), HB2(1), HB3(2), HB4(2), HB5(2), HB6(1), HB7(1), HB8(1)	KT600277-KT600284
	Haiyan county	HY	9	3	0.639	0.00105	HB3(5), HB7(3), HB9(1)	KT600279, KT600283, KT600285
	Huzhu county	HZ	15	3	0.257	0.00077	HB3(13), HB10(1), HB11(1)	KT600279, KT600286, KT600287
	Minhe county	MH	15	5	0.676	0.00127	HB3(8), HB12(1), HB13(4), HB14(1), HB15(1)	KT600279, KT600288-KT600291
	Chengduo county	CD	8	6	0.929	0.00301	HB3(2), HB7(1), HB8(2), HB13(1), HB16(1), HB17(1)	KT600279, KT600283, KT600284, KT600289, KT600292, KT600293
*H. sinense*			52	23	0.867	0.00300		
	Maqin county	MQ	8	2	0.571	0.00083	HS1(4), HS6(4)	KT600294, KT600299
	Guinan county	GN	10	6	0.844	0.00297	HS2(1), HS3(2), HS4(1), HS5(1), HS6(4), HS7(1)	KT600295-KT600300
	Haiyan county	HY	11	10	0.982	0.00438	HS1(2), HS3(1), HS6(1), HS8(1), HS9(1), HS10(1), HS11(1), HS12(1), HS13(1), HS14(1)	KT600294, KT600296, KT600299, KT600301-KT600307
	Tanggula town	TGL	12	9	0.939	0.00345	HS3(2), HS6(3), HS15(1), HS16(1), HS17(1), HS18(1), HS19(1), HS20(1), HS21(1)	KT600296, KT600299, KT600308-KT600314
	Chengduo county	CD	11	4	0.764	0.00174	HS1(4), HS6(4), HS22(1), HS23(2)	KT600294, KT600299, KT600315, KT600316

*N* number of individuals sequenced, *NH* number of different haplotype, *h* haplotype diversity, π nucleotide diversity; The number of individuals observed for each haplotype is given in parentheses

### DNA extraction, amplification, cloning, and sequencing

The third-stage fly larvae were longitudinally cut to retrieve the internal organs. The genomic DNA was extracted from 10 mg of each internal organ using a commercial kit (TIANamp Genomic DNA Kit, TIANGEN Biotechnology, Beijing, China) in accordance with the manufacturer’s recommendations.

We used the primers UEA7 (5’-TACAGTTGGAATAGACGTTGATAC-3’) and UEA10 (5’-TCCAATGCACTAATCTGCCATATTA-3’) to amplify a partial DNA fragment of the COI gene [10]. Each PCR (25 μL) was performed in a PCR tube that contained 1.0 μL of each primer (0.4 μM), 8.5 μL of ddH_2_O, 12.5 μL of *Taq* PCR Master Mix (Sangon Biotechnology, Shanghai, China), and 2 μL of DNA sample in a thermocycler (BIO-RAD, Hercules, USA). The cycling conditions used for PCR were 94 °C for 4 min (initial denaturation), 94 °C for 30 s (denaturation), 55 °C for 1 min (annealing), 72 °C for 1 min (extension) for 35 cycles, and a final extension at 72 °C for 10 min. A negative control (without DNA template) was included in each amplification run. Each amplicon (5 μL) was examined by 1.0 % (w/v) agarose gel electrophoresis to demonstrate amplification efficiency. The PCR products were purified using a DNA Agarose Gel Extraction Kit (Omega, Brattleboro, USA). The purified fragments were cloned into pMD™19-T vector and subsequently transformed into *Escherichia coli* DH5α (TaKaRa, Dalian, China). The recombinant plasmid DNA was obtained and then sequenced using an ABI 3730 DNA sequencer at Sangon Company (Shanghai, China).

### Population haplotype diversity analysis

COI sequences were aligned using MEGA 5.2 [18]. Identical haplotypes were collapsed using DNASP 5.10 [19]. The number of haplotypes and standard diversity indices [haplotype and nucleotide diversities (*h* and π, respectively)] were calculated using DNASP 5.10 [19] for each population.

### Phylogenetic analysis and haplotype network construction

Phylogenetic relationships of *H. bovis* and *H. sinense* COI haplotypes were inferred using Bayesian inference (BI). We selected the best-fit model (GTR + I + G) for BI analyses for each data partition using Modeltest 3.7 [20] in conjunction with PAUP 4.0b10 [21]. A Bayesian tree was constructed using MrBayes 3.1.2 [22], and Markov chain Monte Carlo was run for 10 million generations with sampling every 1000 generations. The first 25 % of generations were discarded as burn-in, and the remaining trees were used to estimate Bayesian posterior probabilities (PP).

COI sequences of *H. bovis* (AF497761) and *H. sinense* (AY350769) obtained from the GenBank database were used for phylogenetic analysis of the species in this study, and COI sequences from three other species of *Hypoderma*, *H. lineatum* (AF497762), *H. tarandi* (AF497764) and *H. actaeon* (AF497765), were used as in-group taxa for the phylogenetic analysis. *Gasterophilus pecorum* (AF497776) was selected as the out-group taxon to root the phylogenetic trees. Median-joining networks of all *H. bovis* and *H. sinense* haplotypes in this study were constructed using Network 4.6 [23] to visualize relationships among unique haplotypes.

### Population genetic and demographic history analyses

Analysis of molecular variance (AMOVA) was used to evaluate *H. bovis* and *H. sinense* population genetic structure in Arlequin 3.11 with 1000 permutations [24]. This study implemented two levels of AMOVA for intra- and inter-population analyses (*Φ*_ST_). Phylogeographic structure of *H. bovis* and *H. sinense* populations was estimated using PERMUT (http://www.pierroton.inra.fr/genetics/labo/Software/Permut) with 1000 permutations. PERMUT tested phylogenetic structure by calculating G_ST_ (only haplotype frequencies) and N_ST_ (differences among haplotypes); phylogenetic structure is usually considered present when N_ST_ is higher than G_ST_ [25]. Fu’s *Fs* and Tajima’s *D* neutrality tests, and sum of squared deviation (SSD) and Harpending’s raggedness (*rg*) test statistics of mismatch distributions were calculated to detect evidence of past population expansions in Arlequin 3.11 [26–28]. In addition, mismatch distributions of *H. bovis* and *H. sinense* populations were performed using DNASP 5.10 [19] to test whether demographic processes were consistent with the mismatch distribution test statistics. A population usually exhibits a uni-modal mismatch distribution when it has passed through a recent demographic expansion [29], whereas a multimodal mismatch distribution indicates that a population is comparatively stable [30].

Expansion time was estimated using the expectation τ = 2 *ut* [31], where τ represents the mode of the mismatch distribution, *t* represents time in generations since expansion, and *u* = 2 μ*k*, where μ is the mutation rate (COI was estimated to be approximately 2 % per million years for *Hypoderma* spp. ) [7] and *k* is the length of the sequence [32]. The *H. bovis* and *H. sinense* generation time was estimated to be 1 y based on their life-cycle [33].

### Divergence time estimates

Divergence times were estimated using the Bayesian phylogenetic method implemented in BEAST 1.8.0 [34]. The clock model was set to relaxed, uncorrelated lognormal [35], with a Yule speciation tree model. Because there is a lack of *Hypoderma* fossils, a secondary calibration approach was used. Based on previous research on divergence time in *Hypoderma* spp.  [10], a mean of normal distribution with standard deviation were set as 8.2 million years ago (Mya) with 0.5 Mya for *H. tarandi* and *H. actaeon*, and 4.2 Mya with 0.5 Mya for *H. bovis* and *H. lineatum*, respectively. The Markov chain Monte Carlo chain length was set to 10 million generations and sampled every 1000 generations. Chain convergence was assessed to determine effective sample sizes greater than 200 for all parameters using Tracer 1.5 [36], and trees were summarized using TreeAnnotator 1.8.1 [34].

## Results

### Sequence variation and haplotype diversity

There were no insertions or deletions of nucleotides in the 689-bp COI sequences amplified from any *H. bovis* and *H. sinense*. A total of 19 nucleotide polymorphisms (12 singleton variable sites and seven parsimony-informative sites) for *H. bovis* and 31 nucleotide polymorphisms (24 singleton variable sites and seven parsimony-informative sites) for *H. sinense* were detected. In total, 17 haplotypes were detected in *H. bovis* populations and 23 haplotypes were detected in *H. sinense* populations (Table 1). Sequences of all haplotypes have been deposited in the GenBank under accession numbers KT600277-KT600316. The ratio of haplotypes relative to the total number of individuals sampled for each species was 0.28 for *H. bovis* and 0.44 for *H. sinense*. There was no significant difference in the number of haplotypes found in each sampling location between the two species (*F*_1, 8_ = 0.459, *P* = 0.517; Table 1). Values of *h* and π were 0.738 and 0.00202 for *H. bovis,* and 0.867 and 0.00300 for *H. sinense*, respectively, which indicates rich genetic diversity in *H. bovis* and *H. sinense* populations; however, there were no significant differences between the two species (*F*_1, 8_ = 0.897, *P* = 0.371; *F*_1, 8_ = 0.809, *P* = 0.395; Table 1).

### Phylogenetic analyses

**Fig. 2:**
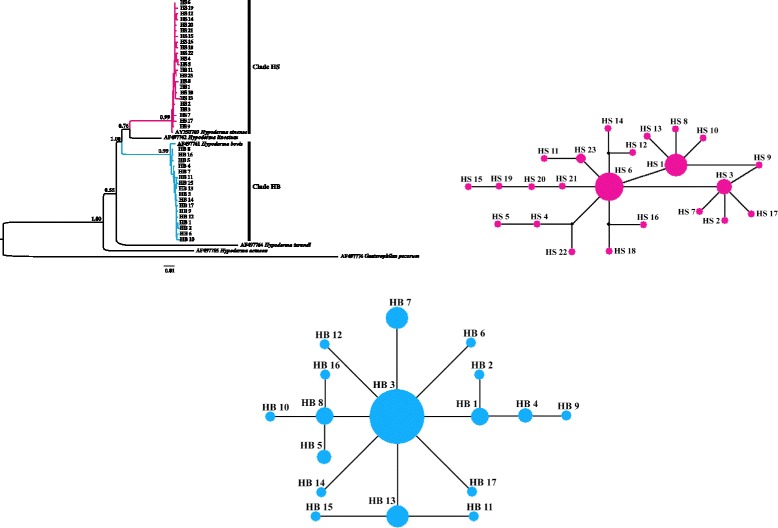
The partitioned Bayesian phylogenetic tree and network of haplotypes for *H. bovis* and *H. sinense* based on COI haplotypes. (*Left*) Bayesian haplotypes phylogenetic tree of *H. sinense* and *H. bovis* based on COI gene sequences. Numbers on branches are Bayesian posterior probabilities. The black bars on the right indicate the corresponding clade HS and clade HB , and each clade (clade HS and clade HB) was presented with different colors. (Right) Median-joining network of haplotypes for *H. sinense* and *H. bovis*. The relative size of circles in the network is proportional to haplotype observed frequencies. The small black dots indicate hypothetical missing haplotypes. The haplotype colors correspond to *H. sinense* and *H. bovis* in phylogenetic tree on the left. All haplotypes designations are listed in Table 1

Bayesian analyses showed that all *H. bovis* haplotypes formed a single well-supported clade designated as clade HB (PP = 0.99; Fig. 2), whereas all *H. sinense* haplotypes formed another well-supported clade designated as clade HS (PP = 0.99; Fig. 2).

The network for clade HB showed that Haplotype HB3 was considered the central haplotype, to which a large number of private haplotypes (76.5 % of the total haplotypes of clade HB) were connected in a star-like manner (Fig. 2). The highest-frequency haplotype was HB3, followed by HB7, HB13, and HB8, which included 30, 5, 5, and 3 individuals, respectively. For clade HS, Haplotype HS6 was considered the central haplotype. The private haplotypes represented 87.0 % of all clade HS haplotypes. The highest-frequency haplotype was HS6, followed by HS1 and HS3, which included 16, 10, and 5 individuals, respectively, and they occupied a central position in the network (Fig. 2).

### Genetic differentiation and population structure

AMOVA results showed that there was significant genetic differentiation in *H. bovis* and *H. sinense* populations (Table 2). For *H. bovis*, AMOVA showed that 11.88 % of the variation was among populations and 88.12 % was within populations. High genetic structure was found (*Φ*_ST_ = 0.119, *P* < 0.001), which indicates remarkable genetic differentiation in *H. bovis*. For *H. sinense*, AMOVA showed that 7.83 % of the variation was among populations and 92.17 % was within populations. We also detected high genetic structure (*Φ*_ST_ = 0.078, *P* < 0.001), which likewise indicates significant genetic differentiation, in the *H. sinense* isolates. Large pairwise *F*_ST_ values were found between *H. bovis* and *H. sinense* populations. For *H. bovis*, pairwise *F*_ST_ ranged from 0.009 to 0.190, and most pairwise values were statistically significant (Table 3). For *H. sinense*, pairwise *F*_ST_ ranged from − 0.037 to 0.210, and most pairwise values were also statistically significant (Table 3).

**Table 2:** Analysis of molecular variance (AMOVA) of COI data from the populations of the two *Hypoderma* species

Source of variation	*H. bovis*				*H. sinense*			
	*df*	*SS*	%	Fixation index	*df*	*SS*	%	Fixation index
Among populations	4	6.500	11.88		4	7.260	7.83	
Within populations	55	34.467	88.12	Φ_ST_ = 0.119***	47	45.374	92.17	Φ_ST_ = 0.078***

*df* degree of freedom, *SS* sum of squares, *%* percentage of variation, Φ_*ST*_ fixation index; ***P < 0.001

**Table 3:** Pairwise *F*_ST_ values among populations for *H. bovis* and *H. sinense* using COI sequences

*H. bovis*						*H. sinense*					
PA	MQ	HY	HZ	MH	CD	PA	MQ	GN	HY	TGL	CD
MQ	0.000					MQ	0.000				
HY	0.095*	0.000				GN	0.210*	0.000			
HZ	0.119*	0.127*	0.000			HY	−0.034	0.116*	0.000		
MH	0.152*	0.176*	0.067	0.000		TGL	0.123*	0.016	0.095*	0.000	
CD	0.009	0.142*	0.190*	0.171*	0.000	CD	−0.037	0.146*	0.003	0.092*	0.000

*PA* population abbreviation; *P < 0.05

**Fig. 3:**
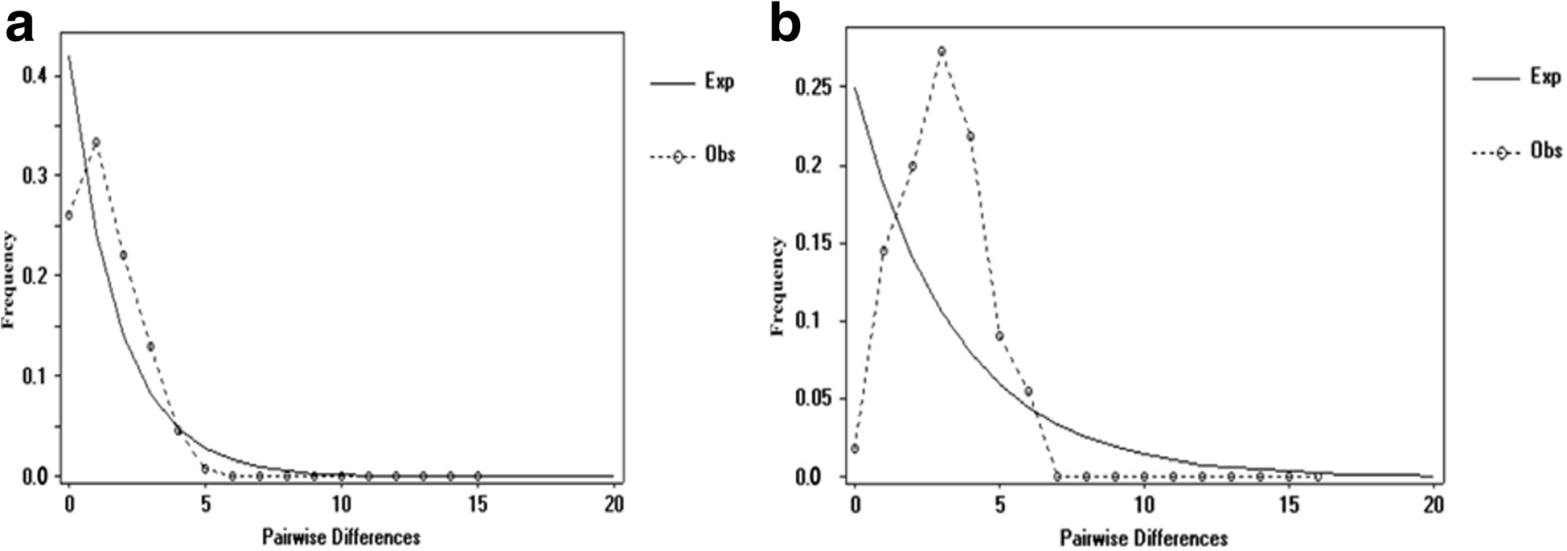
Mismatch distribution analysis for total populations of *H. bovis* and *H. sinense* using DNASP 5.10. Graphs of the mismatch distributions of (**a**) *H. bovis* populations and (**b**) *H. sinense* populations. The X axis shows the observed distribution of pairwise nucleotide differences, and the Y axis shows the frequencies. The dotted lines with circles represent the observed frequency of pairwise differences, and the solid lines show the expected values under the sudden population expansion model

Demographic expansions were analysed for *H. bovis* and *H. sinense* populations using two neutrality tests and mismatch distributions. For *H. bovis* and *H. sinense*, Fu’s *Fs* and Tajima’s *D* values were significantly negative (Table 4), and mismatch distributions of both species each showed a unimodal curve (Fig. 3). From the τ value (2.652) calculated by Arlequin, expansion of *H. bovis* populations was estimated to have occurred about 0.049 Mya; expansion of *H. sinense* populations (τ = 3.098) occurred about 0.056 Mya. A permutation test showed that N_ST_ (0.141) was significantly higher than G_ST_ (0.048) for *H. sinense* (*P* < 0.05), whereas N_ST_ (0.116) was less than G_ST_ (0.122) for *H. bovis*, which indicates that significant phylogeographic structure is apparent in *H. sinense*, but not *H. bovis*.

**Table 4:** Results of the neutrality tests calculated and values of the mismatch distribution test statistics for *H. bovis* and *H. sinense* based on COI sequences

Species	Fu’s *FS*	Tajima’s *D*	SSD	*rg*
*H. bovis*	−12.795***	−2.031 **	0.001	0.035
*H. sinense*	−19.809***	−2.320**	0.001	0.033

*Fu’s FS* Fu’s *Fs* test statistic, *Tajima’s D* Tajima’s *D* Test statistic, *SSD* sum of squared deviation, *rg* Harpending’s raggedness statistic; **P < 0.01; ***P < 0.001

### Divergence times

**Fig. 4:**
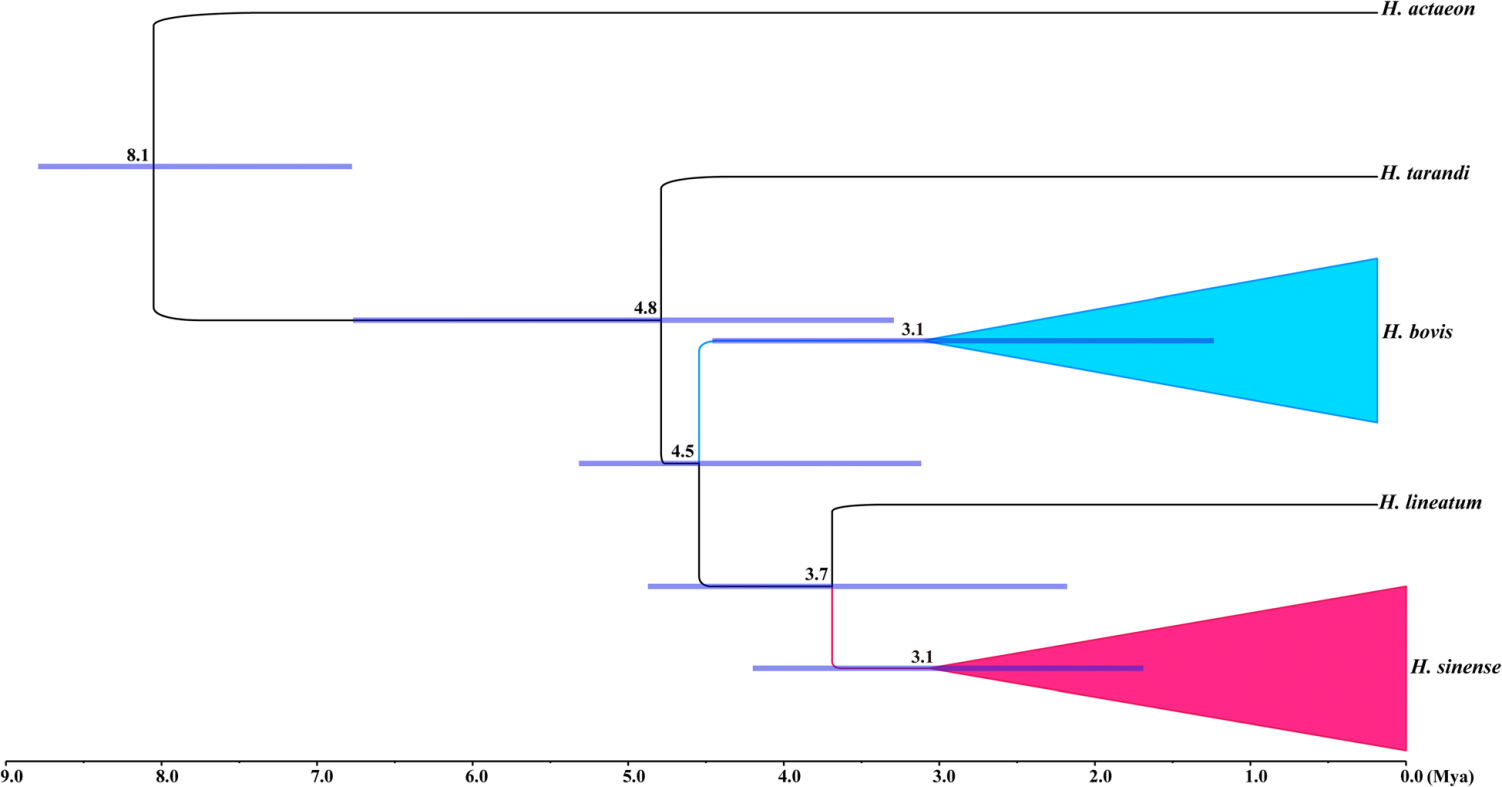
Divergence times in five *Hypoderma* spp. estimated from the COI haplotypes with BEAST. The numbers above nodes are the mean divergence times. The node bars indicated the 95 % highest posterior densities of the divergence time estimates. *H. sinense* and *H. bovis* populations collapsed into their species. A time scale in millions of years is shown below. Outgroup has been removed. For all haplotypes informations, see Table 1

The estimated evolutionary timescale of five *Hypoderma* species with the 95 % highest posterior densities (95 % HPD) intervals are presented in Fig. 4. Our analysis estimated that the most recent common ancestor of the five *Hypoderma* species existed approximately 8.1 Mya (95 % HPD: 6.8–8.8 Mya). The split between two main clades of the three *Hypoderma* species (*H. bovis*, *H. lineatum*, and *H. sinense*) occurred about 4.5 Mya (95 % HPD: 3.1–5.3 Mya), and the divergence time between *H. sinense* and *H. lineatum* was approximately 3.7 Mya (95 % HPD: 2.2–4.9 Mya). Intraspecific divergence times all occurred within 3.1 Mya for *H. bovis* and *H. sinense*.

## Discussion

The haplotype (*h*) and nucleotide (π) diversity are important indicators of genetic diversity in a population [19]. The results showed high *h* and π in *H. bovis* and *H. sinense* populations, which indicates rich genetic diversity of populations of the two species and might explain why the two species have a broad tolerance to environmental and habitat stresses; fast mutational processes inherent in individuals and populations may enable these two *Hypoderma* spp.  to successfully adapt to complex environments.

The Bayesian phylogenetic analysis strongly supported the coalescence of COI haplotypes within *H. bovis* and *H. sinense* (*PP* = 0.99; Fig. 2), which supports the notion that these are different species and is consistent with of the findings of a previous molecular study [8]. In this tree, *H. sinense* and *H. lineatum* (*PP* = 0.76; Fig. 2) were more closely related to each other than to *H. bovis*. This result was not consistent with that of a previous molecular study [37]. This is probably due to the use of different molecular markers and phylogenetic analysis methods. Therefore, more molecular markers and phylogenetic analysis methods should be applied in future studies to resolve this inconsistency. The median-joining networks showed that private haplotypes of *H. bovis* and *H. sinense* were derived from dominant haplotypes (Fig. 2), and the percentages of private haplotypes were high (*H. bovis*: 76.5 % of total haplotypes; *H. sinense*: 87.0 % of total haplotypes), which indicates that populations of the two *Hypoderma* species were closely related respectively, and speciation within *Hypoderma* might be relatively complex.

AMOVA indicated that genetic structure was substantially higher within than among populations (within populations: 88.12 % for *H. bovis* and 92.17 % for *H. sinense*; among populations: 11.88 % for *H. bovis* and 7.83 % for *H. sinense*). Therefore, the majority of *H. bovis* and *H. sinense* genetic differentiation was intra-population. This result may be caused by gene flow. QTP uplift, which resulted in topography changes [38], limited gene flow among the populations, and might have led to gene flow that primarily occurred within the populations; this was likely exacerbated because *Hypoderma* species cannot fly long distances, and adults live for a very short time (only 5–6 days) [39]. Genetic structure among populations was significant (*P* < 0.001) for *H. bovis* and *H. sinense* based on AMOVA (Table 2), which indicates that significant population differentiation occurred between populations of these two species. *F*_ST_ is used to assess genetic differentiation among closely related populations [40]. Our assessment of population genetic structure using the *F*_ST_ index revealed that the range of pairwise *F*_ST_ values is narrower in *H. bovis* (0.009 to 0.190) than in *H. sinense* (−0.037 to 0.210), indicating greater genetic differentiation among *H. sinense* than in *H. bovis* populations. In addition, permutation test showed that *H. sinense* had greater geographic structure of *h* than *H. bovis*. Geographic structure of natural populations is determined by many factors, such as life history, population size, ecological traits, habitat, and historical events [41, 42]. Our results may be caused, in part, by inconsistent habitat in this study for *H. bovis* and *H. sinense*. However, geographic genetic structure was previously shown to negatively correlate with dispersal abilities [43]. In the present research, *H. sinense* exhibited greater geographic genetic structure than *H. bovis*, indicating that dispersal ability is higher in *H. bovis* than *H. sinense*. This finding is consistent with the life history of these species; *H. bovis* mainly parasitize cattle and yaks in plains, hills, and plateaus, whereas *H. sinense* mainly attack bovines in plateau settings [1]. Overall, these results indicate that genetic structure may differ between *Hypoderma* species with differences in habitat preference and dispersal ability.

In this study, the neutrality tests were significantly negative for *H. bovis* and *H. sinense* populations, which indicates that population expansion events may have occurred in the demographic history of *H. bovis* and *H. sinense*. Additionally, the mismatch population test statistics *rg* and SSD for both *H. bovis* and *H. sinense* populations were small and not statistically significant; this indicates that the sudden expansion model, which corresponded with a unimodal curve of the mismatch distribution analysis, could not be rejected (Fig. 3). Overall, these analyses indicated that a demographic expansion had occurred in the *H. bovis* and *H. sinense* populations. τ values reflected estimated expansion times of 0.049 Mya for *H. bovis* and 0.056 Mya for *H. sinense*, which correspond to the late Pleistocene [44, 45]. These expansions probably occurred because *H. bovis* and *H. sinense* populations on the Tibetan Plateau experienced geological changes and climatic oscillations during the QTP uplift and Quaternary glaciation, which may have also led to population differentiation of *H. bovis* and *H. sinense.* After QTP uplift and Quaternary glaciation, many private haplotypes may have been derived from the dominant *H. bovis* and *H. sinense* haplotypes during expansion phases, which resulted in the star-like haplotype networks (Fig. 2).

The COI gene is a global molecular clock gene [46]. In this study, BEAST analyses of COI results estimated that the three *Hypoderma* species (*H. bovis*, *H. lineatum*, and *H. sinense*) diverged approximately 4.5 Mya, which indicates a late Miocene-Pliocene split among *H. bovis*, *H. lineatum* and *H. sinense* [47, 48]. Climatic changes during the late Miocene-Pliocene might have played an important role in the divergence of *Hypoderma* spp.  Furthermore, the divergence time between *H. sinense* and *H. lineatum* was estimated to be 3.7 Mya, which mainly corresponded to the rapid uplift of the QTP approximately 3.6 Mya [49] and therefore indicates that rapid uplift of the QTP could have greatly influenced the divergence of *Hypoderma* spp. More importantly, the rapid uplift of the QTP changed topography, strengthened the East Asia monsoon, and modified global climate [50–52], which may have led to the divergence and speciation of many organisms [53–55]. Therefore, our results indicate that the rapid uplift of the QTP led to habitat isolation, mutation accumulation, and fragmentation in *Hypoderma* populations, and eventually caused speciation of *H. bovis*, *H. lineatum* and *H. sinense.*

*Hypoderma* spp. play a critical role in production losses and susceptibility of cattle and yaks to disease [7, 56, 57]. Our study may be a first step toward a better understanding of *Hypoderma* evolutionary history and speciation, and provides important information for the future study of epidemiological surveillance and hypodermosis control on the QTP.

## Conclusions

This is the first characterization of the genetic diversity and population structure using mitochondrial COI sequences of *H. bovis* and *H. sinense* populations in Qinghai Province, China. The results support the distinction of the two species of *Hypoderma* based on genetic diversity and divergence. Most genetic differentiation of *H. bovis* and *H. sinense* was found within populations, which may have been caused by QTP uplift and life history of the species. Further research including more molecular markers, increased sampling, and different phylogenetic analysis methods are necessary to elucidate genetic differentiation of *Hypoderma* spp. in more detail. In addition, the current findings provide fundamental evolutionary information regarding *H. bovis* and *H. sinense*. These findings also provide a molecular baseline for the control and elimination of *Hypoderma* spp. on the QTP.

## Abbreviations

BI: Bayesian inference
COI: Cytochrome *c* oxidase subunit I
*h*: Haplotype diversity
MCMC: Markov chain Monte Carlo
Mya: Million years ago.
PP: Posterior probabilities
QTP: Qinghai-Tibetan plateau
π: Nucleotide diversity
